# Contraception need and available services among incarcerated women in the United States: a systematic review

**DOI:** 10.1186/s40834-020-00105-w

**Published:** 2020-03-17

**Authors:** Mishka S. Peart, Andrea K. Knittel

**Affiliations:** 1 Clinical Fellow in Complex Contraception and Family Planning, Division of Family Planning, Department of Obstetrics and Gynecology, University of North Carolina at Chapel Hill, Chapel Hill, North Carolina USA; 2 Division of General Obstetrics and Gynecology, University of North Carolina at Chapel Hill, 3027 Old Clinic Building, CB#7570, Chapel Hill, NC 27599-7570 USA

**Keywords:** Contraception, Abortion, Incarceration, Systematic review

## Abstract

**Context:**

Seventy-five percent of incarcerated women are of reproductive age, most of whom are at-risk for unintended pregnancy. Women who are incarcerated come disproportionately from socioeconomically disadvantaged backgrounds and often lack access to desired reproductive health care. While the carceral system provides a unique opportunity to fill this gap, a better understanding of the contraceptive needs, desires, and plans of incarcerated women is needed to optimize health care provision within the carceral system. A review of current contraceptive services available to women inmates may both identify model care programs and shed light on areas for improvement.

**Evidence acquisition:**

PubMed electronic database used to identify relevant articles published between January 1975 and September 2019 using a systematic review method.

**Results:**

Twenty-five articles met the inclusion criteria and answered four key questions surrounding contraception in the carceral system. Most articles (48%) represented scientific research. Other publications identified by this review were expert commentaries, policy briefings, guidance and recommendations reports, and law and bioethics reviews.

**Conclusions:**

Incarcerated women desire access to standard and emergency contraception from carceral health care systems. Knowledgeable family planning practitioners providing patient-centered and trauma-informed care and public health interventions linking newly released inmates to community clinics can help alleviate inmates’ concerns regarding initiating desired contraception while incarcerated.

## Introduction

According to the U.S. Department of Justice, more than 200,000 women were imprisoned in the United States in 2015, with approximately an equivalent number of women detained in jail facilities either pre-trial or serving sentences [1, 2]. Across prison systems, the overwhelming majority are housed in state carceral systems (189,800), and 12,900 residing under federal care. Three-fourths of incarcerated women are of childbearing age at the time of intake [3], and 6–10% of women are pregnant at the time of incarceration [4].

Because the incarceration rate for women continued to increase exponentially over the past three decades and most incarcerated women are between the ages of 18–44 years old [2], the carceral health care system is in a unique role to address the reproductive needs of its residents.

Although many individuals may use the terms “jail” and “prison” interchangeably, there are important distinctions between the two. Jail is a confinement facility where people stay while awaiting trial or sentencing or serve short sentences and is run by local law enforcement. The length of stay in jail very rarely exceeds 1 year and inmate turnover tends to be high. Prison, on the other hand, is generally for individuals who have already been convicted of a crime and received a sentence. The prison length of stay tends to be longer with less turnover. Prisons are run by the state or federal government or private companies that are contracted with the government. We use the phrase “carceral system” as an inclusive term encompassing both jails and prisons.

The carceral system represents the only health care safety net available for many women to receive the care they need or desire, including access to contraceptive services. Both the American Public Health Association and the National Commission on Correctional Health Care endorse that contraceptive services should made available to women as part of carceral care [5, 6]. The American College of Obstetrics and Gynecology also support that incarcerated women of all ages have access to reproductive health care including contraception, prenatal care, and abortion [4]. Even with the support of national organizations, there are barriers limiting the contraceptive care women receive, including women’s apprehension about reproductive health care in a carceral context due to historical personal or collective injustices against incarcerated women.

Knowledge regarding the level of contraceptive need, the services incarcerated women desire, and the types of programs currently available is required for a better understanding of how to advocate for and serve these women. The aim of this review was to identify current contraceptive services available to women in carceral facilities, to describe attributes of model care programs, and to shed light on areas for improvement.

## Methods

We conducted a systematic search of the published literature including research articles, commentary works, guidelines and recommendations, law reviews, and policy briefings using the major online research literature database, PubMed. Our review process began with the development of four key questions (Table 1) on which to focus our search. Retrieval and inclusion criteria were determined a priori and applied to search results. In consultation with a medical research librarian, we developed the PubMed search syntax below:

**Table 1:** Key questions for systematic review on contraception and abortion services among incarcerated women in the United States

Key Question No.	Question	Publications addressing the Key Question
1	What is the contraceptive need among incarcerated women?	1. Clarke et al., 2006^a^ 2. Clarke et al., 2006^b^ 3. Clarke et al., 2006^c^ 4. Hale et al., 2009 5. LaRochelle et al., 2012 6. Cannon et al., 2018 7. Ghidei, Ramos, Brousseau, & Clarke, 2018
2	Can incarcerated women access contraceptive and abortion services?	1. Fielder & Tyler, 1975 2. Kasdan, 2009 3. Sufrin, Creinin, & Chang, 2009^d^ 4. Sufrin, Creinin, & Chang, 2009^e^ 5. Roth, 2011 6. Kouros, 2013 7. Kraft-Stolar, 2015 8. Roth & Ainsworth, 2015 9. Sufrin, Kolbi-Molinas, & Roth, 2015 10. Sufrin, Oxnard, Goldenson, Simonson, & Jackson, 2015 11. American Civil Liberties Union of California, 2016 12. Knittel, Ti, Schear, & Comfort, 2017 13. Roth, 2017 14. Sufrin, Baird, Clarke, & Feldman, 2017 15. Sufrin C., 2019
3	What contraceptive services do incarcerated women want?	1. Clarke et al., 2006^c^ 2. Sufrin, Tulsky, Goldenson, Winter, & Cohan, 2010 3. LaRochelle et al., 2012 4. Schonberg, Bennett, Sufrin, Karasz, & Gold, 2015 5. Cannon et al., 2018
4	What reproductive and contraceptive plans do incarcerated women have after release from correctional facilities?	1. Hale et al., 2009 2. Oswalt et al., 2010 3. LaRochelle et al., 2012

Author Jennifer G. Clarke published several articles in 2006 which were included in our analysis. These articles are noted throughout the remainder of the manuscript as the following:

^a^ Reproductive Health Care and Family Planning Needs Among Incarcerated Women [7]

^b^ Pregnancy and Contraceptive Attitudes Among Women Entering Jail [8]

^c^ Improving Birth Control Service Utilization by Offering Prerelease Vs. Postincarceration [9]

Author Carolyn B. Sufrin published two articles in 2009 which were included in our analysis. These articles are noted throughout the remainder of the manuscript as the following:

^d^ Contraceptive Services for Incarcerated Women: A National Survey of Correctional Health Providers [10]

^e^ Incarcerated Women and Abortion Providers: A Survey of Correctional Health Provider [11]

(gynecolog* OR reproductive OR OBGYN[tw]) AND (woman* OR women* OR female*) AND (“Prisoners”[MeSH] OR jail[tw] OR jailed[tw] OR prison*[tw] OR imprison*[tw] OR convict*[tw] OR felon*[tw] OR incarcerat*[tw] OR correctional[tw] OR inmate*[tw]) NOT (cichlid OR “incarcerated uterus”) AND (contracept* OR LARC* OR abortion* OR sterilization* OR Depo OR inject* OR pill OR patch OR ring OR IUD OR IUC OR implant OR Nexplanon OR intrauterine*)

**Fig. 1:**
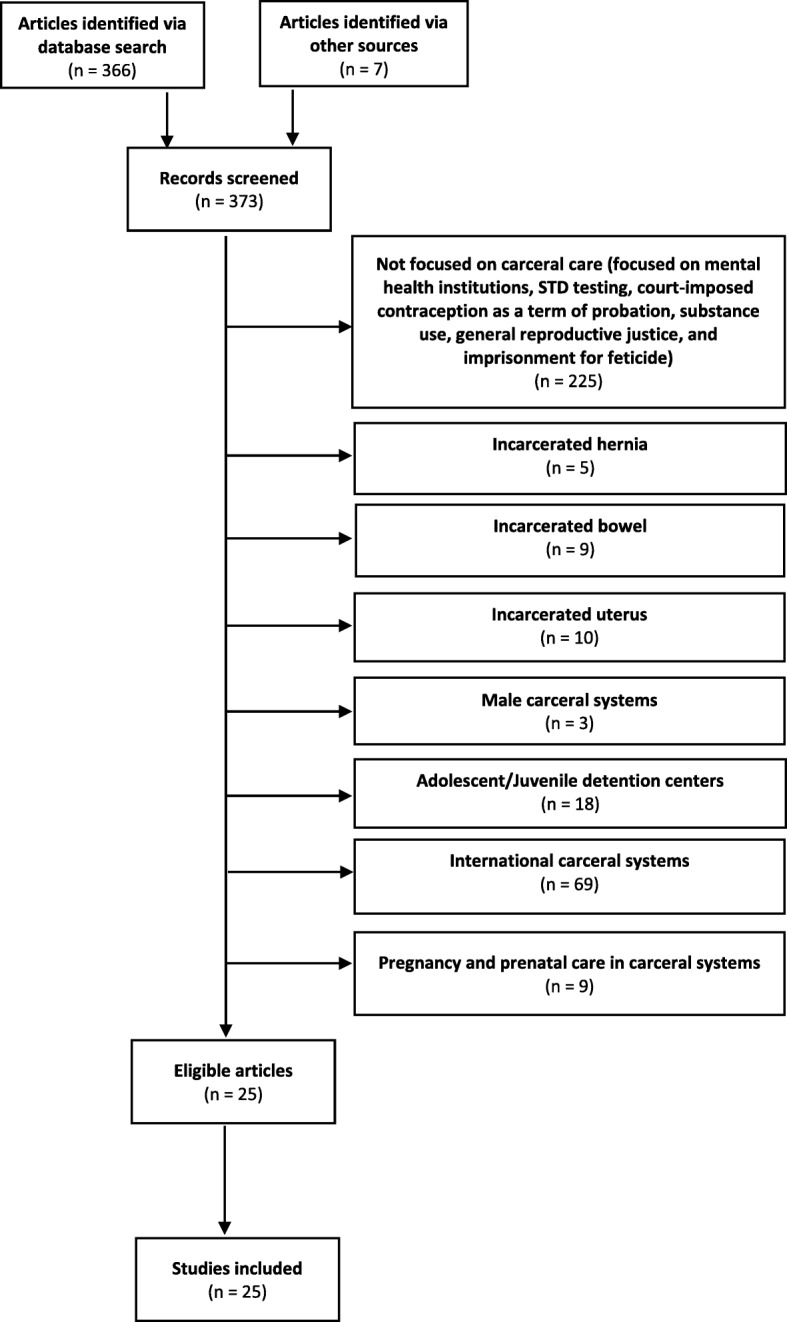
Flow chart demonstrating study selection process

For completeness, we also searched literature known to the authors for evaluation for inclusion. Full-text articles published in English between January 1, 1975 and September 30, 2019 with a focus on contraception and abortion in United States adult women’s carceral systems were eligible for inclusion. Based on this process, we identified 366 articles. We immediately excluded 225 articles, which were not focused on carceral care, but instead on mental health institutions, STD testing, court-imposed contraception as a term of probation, substance use, general reproductive justice, and imprisonment for feticide. We then excluded articles regarding incarcerated bowel, hernia, or uterus (*n* = 24), male carceral systems (*n* = 3), adolescent and juvenile detention centers (*n* = 18), international carceral systems (*n* = 69), and pregnancy and prenatal care in the carceral system (*n* = 9). This screening process led to the identification of 25 eligible articles for inclusion in the study. Figure 1 summarizes the review process.

For simplification, this review uses the terms “woman”, “women”, and female pronouns, reflecting the language used in the included publications. We recognize that there are individuals who may not identify as women but are still able to become pregnant and may desire contraception.

## Results

A total of 25 articles were selected for this review, ranging in date of publication from 1975 to 2019. Articles that addressed more than one key question were included in each category and were not considered exclusively to address a single question. A summary of the different types of publications included in this review can be found in Table 2, with scientific research comprising almost half of all articles. These studies included both qualitative and quantitative analyses in the form of cross-sectional survey data, semi-structured interviews, and retrospective analyses.

**Table 2:** Article types included in review

Article type	Frequency	%
Scientific research	12	48%
Commentary	5	20%
Policy briefing	3	12%
Guidance and recommendations	2	8%
Law review	2	8%
Bioethics review	1	4%
Total	25	100

### Key question 1: what is the contraceptive need among incarcerated women?

The seven articles identified addressing the contraceptive need among incarcerated women can be found summarized in Table 3. Three articles (43%) reported information on jails only, in contrast to the remaining four that reported data from an integrated jail and prison correctional system. The articles reporting jail data only are noted as such.

**Table 3:** Summary describing the contraceptive need in American women’s carceral system (key question 1)

Reference and Year	Describes prevalence of unintended pregnancy	Evaluates abortion prevalence	Assesses preincarceration contraception utilization	Evaluates desire to use contraception	Reports prevalence with access to health-care provider	Estimates who is at risk for pregnancy post-release ^a^
Clarke et al., 2006^b^	↑	↑	↑			↑
Clarke et al., 2006^c^	↑	↑	↑	↑		↑
Clarke et al., 2006^d^	↑	↑	↑			
Hale et al., 2009			↑	↑	↑	↑
LaRochelle et al., 2012		↑	↑	↑	↑	
Cannon et al., 2018		↑	↑	↑		↑
Ghidei, Ramos, Brousseau, & Clarke, 2018	↑					
Total (%)	4/7 (57)	5/7 (71)	6/7 (86)	5/7 (71)	2/7 (29)	2/7 (29)

^a^ At-risk for pregnancy is defined as women of reproductive age with a uterus, having intercourse with men and not already using a highly effective form of contraception as defined by the World Health Organization (intrauterine device, subdermal implant, or tubal sterilization)

^b^ Reproductive Health Care and Family Planning Needs Among Incarcerated Women [7]

^c^ Pregnancy and Contraceptive Attitudes Among Women Entering Jail [8]

^d^ Improving Birth Control Service Utilization by Offering Prerelease Vs. Postincarceration [9]

Clarke et al. in 2006 determined that among women in the Rhode Island Adult Correctional Institute, 84% previously experienced an unplanned pregnancy and 35% had a history of at least one abortion [7]. This is considerably higher than nationally reported data, where 45% of US pregnancies were unintended [12] and 24% of US women had had an abortion [13]. Of women within the study who were at risk for pregnancy, only 28% consistently used birth control during the 3 months prior to incarceration and only 20% consistently used a condom, thus increasing the risk of unintended pregnancy at the time of carceral entry. Eighty-five percent of these women at-risk for pregnancy reported that it would be likely for them to have intercourse with a man within 6 months of release.

Clarke and colleagues collected survey data in 2006 on a similar Rhode Island population of prisoners as above [8]. They discovered that 50% of inmates had negative attitudes towards pregnancy (i.e., they did not want to become pregnant). Another 41% of respondents acknowledged ambivalent pregnancy attitudes. Among the women with negative pregnancy attitudes, 91% experienced a prior unintended pregnancy and 40% had a history of abortion. Overall, 55% of the population surveyed reported wanting to start a birth control method immediately, with a greater proportion of those with negative pregnancy attitudes desiring initiation. Preincarceration contraception use was similar to Clarke’s prior study described above, and 42% of respondents perceived some chance of becoming pregnant in the next 6 months. Clarke et al., 2006 demonstrated in another study that almost 80% of incarcerated women desired to initiate contraception while within the correctional facility [9]. They also discovered that women were more likely to initiate contraception if it was provided while in jail or prison (discussed in further detail under key question 3). In this study, 64% of women experienced a prior unintended pregnancy and 34% a prior abortion.

Hale and colleagues recruited respondents from five local jails in the southeast U.S in 2009 [14]. In this study, 62% of reproductively capable women used contraception almost all the time, and 76% planned to have sex after release from jail and were at risk of unintended pregnancy. Of these reproductively capable women, 64% reported access to a provider prior to arrest, with a similar proportion reporting access to a health care provider after jail release. It is important to note that only 25.5% of respondents reported having access to an OB/GYN, which has important implications for the types of contraceptive options that may be offered to them and continued surveillance of their chosen contraceptive method.

In a San Francisco jail population in 2012, LaRochelle et al*.* found that 54% of respondents had a history of abortion; overall 45% of all women sampled wanted to use contraception post-release and 60% would accept it if it were offered from jail health services [15]. An average of 28% reported finding a provider or clinic as a barrier to contraception use, with 52% of women who reported not using contraception prior to incarceration noting this as a barrier. Cannon and colleagues in 2018, in contrast, found that 42% of respondents from Cook County jail had a history of at least one prior abortion and 72% desired contraception that would be offered from the jail health service [16].

Taken together, these studies demonstrate that incarcerated women are at higher risk for unintended pregnancy and abortion and will remain at increased risk for pregnancy post-release due to no or inconsistent contraception use preincarceration and poor access to health care providers. The overwhelming majority desired to use contraception. These findings effectively demonstrate the need for contraception in this population.

### Key question 2: can incarcerated women access contraceptive and abortion services?

Fourteen publications addressing this question were identified. They are listed and summarized in Table 4. This section contains the greatest variation of publication types, including at least one article from each of the six article types listed in Table 2. Except for one article which will be specifically noted, all publications comment on jails and prisons collectively.

**Table 4:** Summary of articles surrounding reproductive service availability in American carceral system (key question 2)

Reference and Year	Describes length of time to access health care provider	Provision of family planning education	Contraception provision and policies	Emergency contraception provision	Access to abortion care and policies	Unbiased pregnancy options counseling	Permanent sterilization
Fielder & Tyler, 1975		↑	↑				
Kasdan, 2009					↑		
Sufrin, Creinin, & Chang, 2009^a^			↑	↑	↑		
Sufrin, Creinin, & Chang, 2009^b^		↑	↑		↑	↑	
Roth, 2011					↑	↑	
Kouros, 2013							↑
Kraft-Stolar, 2015		↑	↑	↑	↑	↑	↑
Roth & Ainsworth, 2015		↑	↑		↑	↑	↑
Sufrin, Kolbi-Molinas, & Roth, 2015			↑		↑	↑	
Sufrin, Oxnard, Goldenson, Simonson, & Jackson, 2015			↑				↑
American Civil Liberties Union of California, 2016		↑	↑	↑	↑	↑	↑
Knittel, Ti, Schear, & Comfort, 2017			↑		↑	↑	
Roth, 2017					↑		↑
Sufrin, Baird, Clarke, & Feldman, 2017		↑	↑	↑			↑
Sufrin C., 2019					↑		
Total (%)	0/15 (0)	6/15 (40)	10/15 (67)	4/15 (27)	11/15 (73)	7/15 (47)	7/15 (47)

^a^ Contraceptive Services for Incarcerated Women: A National Survey of Correctional Health Providers [10]

^b^ Incarcerated Women and Abortion Providers: A Survey of Correctional Health Provider [11]

Fiedler and Tyler in 1975 describe a pilot family planning program to provide education and services to incarcerated women in New York City [17]. The program was limited to education and counseling conducted during the week prior to a woman’s release from prison only. Contraceptive initiation was not allowed due to concern for complications and lack of follow-up. For many New York prisons, contraception provision is still not allowed today. Although this article describes an important movement to provide carceral contraceptive options in this area, the authors pejoratively generalize about the women they serve, stating that they “lack interest in their own health”, and “suffer from self-neglect”.

Kasdan addresses a woman’s right to abortion while incarcerated [18]. While the right to an abortion is not lost as a result of incarceration, certain carceral policies, such as only allowing inmate transport for medically necessary procedures, may delay care and make an abortion increasingly difficult to obtain when it is deemed elective.

Sufrin and colleagues published two studies in 2009a and 2009b exploring correctional care provider responses about contraception services and abortion provision [10, 11]. Thirty-eight percent of respondents reported that birth control and emergency contraception were provided at their facility and while 70% of providers state that some degree of contraception counseling was performed, only 11% of responders provided routine counseling prior to release. As mentioned above, incarceration does not legally restrict a woman’s right to abortion, however in their second study, only 68% of providers surveyed stated that incarcerated women could obtain an abortion. Eighty-eight percent of responders stated that the facility provided transportation, but only 54% of providers stated that they assisted with arranging appointments. This is evidence for additional logistical barriers beyond the legal right to abortion. Many states require mandatory waiting periods varying from 24 to 72 h, mandated abortion counseling content, and restrictions on using public funding for abortion, all of which can delay a woman’s access to abortion care in any context [19]. Based on location, women in carceral systems are subject to these same state restrictions in addition to limitations of their personal liberties such as using the phone or internet to schedule an appointment or calling a clinic for information about a procedure. This may make seeking an abortion from behind bars incredibly difficult and may result in lengthy delays in care.

Roth’s 2011 commentary piece details policies regarding access to abortion care and pregnancy options counseling [20]. She states that one-third of states have policies mandating prison staff to inform women of all their pregnancy options, including abortion. Another one-third of states use conditional wording to provide options counseling only in the event that the woman mentions abortion herself. Some states require that women inmates bear the burden of additional costs to obtain an abortion, such as gas, toll, and wages of the officers that are required when they travel off site. At least eight states have no written policy on abortion, a situation that leaves important decisions in the hands of prison officials. Sufrin joins Roth and Kolbi-Molinas in 2015 to extend this discussion and describe how prison and jail officials who deny incarcerated women access to abortion punish women by forcing them to continue their pregnancies [21].

In a 2013 bioethics review, Kouros discusses the unapproved sterilization of 148 California inmates between 2006 and 2010 [22]. Some women later reported feeling pressured into sterilization. According to the American College of Obstetricians and Gynecologists, incarcerated women should undergo sterilization very rarely, and only after access to LARC methods have been available and excellent documentation of prior (pre-incarceration) request for sterilization is available. These additional safeguards are needed because of the likelihood that the coercive environment of prison hampers true informed consent [23]. The College also states that policies denying all sterilization may encroach upon some women’s genuine desire to be sterilized and should be reconsidered, especially because many women may not have access to sterilization outside of the prison system. Roth and Ainsworth in 2015 completed a law review exploring the history of sterilization of incarcerated women that led to the adoption of federal regulations against the practice [24].

In her detailed report from 2015 on the state of the New York prison system, Kraft-Stolar describes how the carceral system prohibits its providers from prescribing contraceptives with very few exceptions [25]. Women participating in the Family Reunion Program, being released from the prison, or undergoing treatment for hepatitis C (because of the teratogenic nature of antiviral medications) can be provided with condoms only. No other contraception is permitted. This may be particularly problematic for women in the Family Reunion Program and are concerned about asking their partners to use a condom. There was a short period from 2009 to 2013 when the carceral system contracted with Planned Parenthood to offer contraception to women at certain prisons that were within 2 weeks of their release date. However, the funding was cut, and the initiative subsequently ended. The opportunity to participate in a two-hour class about family planning and general health prior to release also ended with the expiration of the program.

Kraft-Stolar continues to outline how contraception is not offered for women in work release programs within the New York prison system, although they spend time in the community and may have sexual partners there. As if the implication of an unintended pregnancy were not significant enough, women who become pregnant may be terminated from their work release program. Many women also reported that they were denied contraception for reasons unrelated to pregnancy prevention (menstrual regulation, dysmenorrhea, etc.) even when prescribed by an outside provider. There were conflicting reports about whether emergency contraception was provided, although review of the prisons noted that emergency contraception was not dispensed within the last decade. The report also states that there is no central written policy on abortion, which as described elsewhere in this paper can be problematic for several reasons. Some women noted standard policies such as those that served disciplinary action to women who made a medical appointment and canceled it, discouraging women from making appointments.

Sufrin, Baird, Clarke, and Feldman’s 2017 publication did list four model programs offering carceral family planning services, one of which is Rikers Island jail in New York [26]. Rikers jail complex stands in contrast to the New York prison facilities described in other publications above, in that there is a policy on contraception provision and all contraceptive options and emergency contraception are available. However, in 2019 New York City lawmakers voted to close the jail, which is scheduled to be shuttered by 2026 with distribution to smaller more “modern” jails located closer to the city’s main courthouses [27]. Other exceptional carceral reproductive care models include Cook County Jail, Rhode Island Department of Corrections, and San Francisco County Jail. Sufrin and colleagues’ retrospective study from 2015 on LARC provision feasibility is the only publication in this section that focused exclusively on a local jail population and found LARC to be a safe and feasible option in this setting [28].

In sum, access to contraception varies across facility types, geography, and programs (e.g. work release, public-private partnerships, etc.), and is limited by concerns about coercion, cost, and a lack of consistent policies. We noted that multiple articles refer to “timely” access to abortion services without specification of a time frame. Similarly, none of the included articles discussed what constituted a reasonable length of time to access a health care provider for a concern or problem visit. Despite this, there is evidence for feasibility of model programs providing the full range of contraceptive options within a carceral setting.

### Key question 3: what contraceptive services do incarcerated women want?

A summary of five articles addressing the question of what contraceptive services do incarcerated women want (key question number three) can be found in Table 5. All articles except the Clarke et al., 2006 [9] publication focus on specific jail populations in Chicago, San Francisco, and New York. Clarke’s paper included women from both jail and prison populations, making the results potentially more generalizable to women in various divisions of the carceral system.

**Table 5:** Summary of contraceptive services women in the American carceral system want (key question 3)

Reference and Year	Contraception provision	Emergency contraception provision	Educational classes	Non-experimental/gold standard care	Trusted providers	Postrelease follow-up care
Clarke et al., 2006^a^	↑					
Sufrin, Tulsky, Goldenson, Winter, & Cohan, 2010		↑				
LaRochelle et al., 2012	↑					
Schonberg, Bennett, Sufrin, Karasz, & Gold, 2015	↑		↑	↑	↑	↑
Cannon et al., 2018	↑	↑				
Total (%)	4/5 (80)	2/5 (40)	1/5 (20)	1/5 (20)	1/5 (20)	1/5 (20)

^a^ Improving Birth Control Service Utilization by Offering Prerelease Vs. Postincarceration [9]

Clarke examined whether contraceptive availability within the carceral system would increase birth control initiation among women who are incarcerated [9]. This study found that almost 80% of respondents reported a desire to initiate contraception during incarceration and that women who were housed in facilities offering contraception were over 14 times more likely to initiate a contraceptive method compared to those who were not. Half of the women chose to use oral contraceptive pills, 48% chose depo medroxyprogesterone acetate injectable, and 2% opted for intrauterine devices. Even when connected with a free clinic post-release for contraceptive provision, only 4.4% of women who reported interest in contraceptive initiation started a method if it was not offered to them while in jail/prison. This suggests that contraceptive provision in the carceral system would be welcomed and well-utilized by women who are incarcerated.

In a 2010 publication, Sufrin and colleagues addressed emergency contraception provision in the jail population [29]. Based on a 63-item survey, they discovered that 29% of women being booked into a San Francisco jail were eligible were emergency contraception services, and half of these women would accept emergency contraception if offered. Over 70% of women who were eligible for emergency contraception had either a negative pregnancy attitude or were ambivalent towards a new pregnancy. Over 40% of these women had experienced a prior abortion. Finally, 71% of all women surveyed stated that they would accept an advance supply of emergency contraception upon release from jail. These findings suggest that newly arrested women are at high-risk for unplanned and unintended pregnancy and emergency contraception provision is not only desired among this population but may have important implications to increase reproductive service access among this traditionally marginalized population and decrease their risk of unintended pregnancy. Larochelle et al., in 2012 found that 60% of all women surveyed in San Francisco desired contraception be available through the jail health services and would accept its use if offered [15]. Additionally, 88% of women who did not access contraception in the year prior to the study but wanted to, stated that they would accept birth control if offered in jail.

In 2015, Schonberg and colleagues published a qualitative study that explored what incarcerated women desired in contraceptive services offered in jail [30]. This was the only qualitative study included in our review. Nearly 100% of women interviewed believed that contraception should be available as a basic health service while in jail. While most felt that all forms of contraception should be available while in jail, a few thought it would be better suited to include in discharge planning- either at the jail or by referral to a local community clinic. One woman explained that she would like to have contraception offered in jail in case it “takes longer than planned to get on [her] feet”. She wanted to ensure that she was protected against pregnancy as she took steps to improve and enhance her life. Other desires that respondents expressed were sexual education classes, counseling, and printed materials.

Cannon and colleagues explored contraceptive desires among women housed at Cook County jail in Chicago [16]. They determined that 73% of respondents were interested in contraceptive supplied if provided free of charge just prior to release and 82% of women were interested in receiving a free supply of emergency contraception.

Across studies, the respondents also explained their apprehensions about utilizing contraception from the jail health care system. The most prevalent concern was about lack of follow-up once released. This was especially true regarding long-acting reversible contraception, which requires provider assistance for discontinuation. Another concern was potential stigma associated with contraceptive use. As explained by respondents, a woman on birth control in a single-sex jail raised suspicions regarding the woman in question having sexual relations with male jail staff. Other concerns included feeling that the products they received would be lesser quality or experimental when compared to care sought outside the carceral system, or that providers were either very early in their training, lacked knowledge, or were too forceful about prescribing birth control methods without taking to time to review side effects or the inmates’ concerns. These concerns may be valuable for those providing carceral health care, jail/prison administrators who make decisions about the type of services offered, and public health officials with an interest in this population.

None of the articles mentioned women’s desires surrounding pregnancy options counseling or abortion care while incarcerated. Although majority of prison pregnancies end in a live birth [3], this population is at high-risk for unintended pregnancy and alternatives to parenting such as adoption services and referrals for abortion should remain available.

All told, women who are incarcerated report a desire to initiate contraceptive methods during incarceration or receive their initial prescription at the time of release, provided that their concerns about provider training, stigma, and community follow-up are addressed.

### Key question 4: what reproductive and contraceptive plans do women who are incarcerated have after release from correctional facilities?

Table 6 summarizes the findings from the three articles addressing women’s plans to become pregnant or use contraception post-release, all of which focus specifically on local jail populations. Hale et al. in 2009 found that 45% of reproductively capable women did not desire to ever have children in the future, and an additional 19% did not desire to become pregnant in the first 2 years post-release [14]. Among the 72.4% of respondents who reported intentions to use birth control with every act of intercourse post-release, 69.1% planned on using the male condom, 15.5% the oral contraceptive pill, 10.3% withdrawal, and 6.2% contraceptive injection. Respondents were also asked about their contraceptive choices if money and availability did not matter, and 4.7% reported that they would pursue tubal ligation and a larger proportion opted for the contraceptive injection. As discussed under key question 1, only 25% of reproductively capable women had access to an OB/GYN prior to incarceration, and only 57% of individuals believed that they would still have access to health care after release.

**Table 6:** Summary of postrelease reproductive and contraception plans (key question 4)

Reference and Year	Defines post-release conception plans	Assesses post-release plans to use contraception	Describes intended methods of contraception	Assesses where women plan to obtain contraception
Hale et al., 2009	↑	↑	↑	↑
Oswalt et al., 2010	↑	↑	↑	↑
LaRochelle et al., 2012	↑	↑		
Total (%)	3/3 (100)	3/3 (100)	2/3 (67)	2/3 (67)

In another population, Oswalt et al. in 2010 found only 38.5% of women desired to become pregnant after release from jail [31]. Among the 62.4% respondents who reported intentions to use birth control with every act of intercourse postrelease, the preference for contraceptive method was similar to the results of the Hale et al. (2009) study. Women who planned on using the male condom made up 69.1% of the respondents, 15.5% planned to use oral contraceptive pills, 10.3% planned to use the withdrawal method, and 6.2% planned on using the contraceptive injection. Non-White women were less likely to use contraception after release vs. White women (67% vs. 80.9%, *p* < 0.05). Furthermore, the study found that only 63.2% of respondents reported that they would have access to a health care provider after release.

Neither the Hale nor Oswalt publications included long-acting reversible contraceptive (LARC) options in their surveys, because their population was drawn from five local jails in the southeast United States that did not provide LARCs at baseline [14, 31]. Therefore, these studies did not identify women who were planning on using LARC methods after jail release. Similar to the Sufrin et al. publication on emergency contraception [29], LaRochelle et al. found that 78% of incarcerated women reported either a negative or ambivalent attitude towards pregnancy, and that 45% of women wanted to use contraception after their release from jail, although they did not include the specific method desired [15].

Taken together, these studies show that women desire a range of contraceptive options after they return to the community, but face barriers related to cost and access to providers for follow-up.

## Discussion

From this review, we have identified not only the contraceptive services that incarcerated women desire, but also potential barriers that limit the uptake of contraception when offered through the carceral care system. Barriers that we found are similar to those that have been described in earlier commentaries, and include a lack of provider training about birth control methods as well as women’s concerns about their ability to continue (or discontinue in the setting of LARCS) their chosen contraceptive method within the community due to cost and access to providers [32].

Women across multiple studies reported concern that providers were either not knowledgeable about contraceptive options or would seldom discuss side effects. Another respondent during a semi-structured interview commented specifically about being cared for by medical trainees. While collaborations with medical and other health professions schools may be an important component of educating students about the health needs of this population, and potentially increasing access of care to incarcerated women, there must be adequate training and supervision for students counseling women about reproductive planning. Incarcerated women may be distrustful of carceral care system due to historical collective or personal injustices but having familiar and knowledgeable family planning practitioners providing patient-centered and trauma-informed care may help alleviate these concerns.

Another barrier to contraceptive use that was identified was follow-up once in the community. Public health interventions such as linkages to clinics through warm hand-offs between carceral systems and community or academic providers or other programs for continued access to contraception may improve access to contraceptive surveillance for women once they return to the community.

The specific type of carceral facility also determines which specific barriers a woman may face to accessing contraceptive care. While most state and federal prisons provide care to prisoners, the availability and access to care in jails is highly variable [4]. The short duration of incarceration also makes care provision difficult. Historically, health care in the carceral system was delivered via a “sick call” model where an inmate actively sought out medical attention. This system does not allow for preventive care and health education. Contraceptive needs would more adequately be met by a model that integrates standard medical exams at the time of intake and regular scheduled health maintenance visits throughout incarceration, with more time for health education compared to problem-focused visits.

Incarceration does not preclude a woman’s constitutional right to abortion, however carceral systems with no written policy on abortion leave much to the interpretation of prison officials. Even when carceral health care workers are supportive of a woman’s right to access abortion, she may have to manage the logistics of coordinating her care with an outside provider and will still face the delays imposed in many states on all women seeking abortion. This is not a simple process for women who are not incarcerated, and having to do so in a facility where the flow of information and the amount of time that one has access to a phone to make appointments is limited and controlled by prison officials may prove extremely challenging and lead to more delays in care. The articles reviewed included reports of some prisons denying a woman an abortion until she is released under the premise that it is an elective procedure. Given that elective cases are defined as those that can be postponed without irreversible or serious harm, this by definition makes abortion a medically necessary case and should be treated and documented as such. Doing otherwise represents the extreme opposite of a patient-centered approach to abortion care, and legal and policy interventions will likely be required to avoid unnecessary delays in care. Also, if inmates are required to coordinate their own care, then they should be presented with a list of supportive family planning organizations and clinics that will assist her.

## Limitations

This review had several limitations. Eight publications (32%) focused specifically on women incarcerated in jails in lieu of prisons. This limits the generalizability of these studies to the prison population, where women may be housed for a longer period of time. Because the average length of stay in jail is relatively short compared to prison inmates, averaging about 25 days [33], this has important implications for the most appropriate model of care to offer services for many women.

All but one scientific publication required that participants be fluent in English in order to participate in the study. The 2009 study by Hale and colleagues did not mention such eligibility criteria [14]. Inmates of Hispanic ethnicity comprised 15% of the prison population in the United States in 2016 and in some states, account for almost 40% of prisoners [33, 34]. Many of these individuals may not be fluent in English. Since a language may serve as a logistical barrier when accessing health care, non-English-speaking women excluded from these studies may potentially represent some of the most marginalized women in the carceral system with unique contraceptive needs and preferences, representing another limitation of this study.

We initially considered including a quantitative approach to our systematic review, such as a meta-analysis, in addition to a qualitative summary, but the diverse methodologies and study designs employed in the articles we identified did not permit this. Additionally, our decision to focus our review on contraception among incarcerated adult women, the information presented should not be generalized to juvenile/adolescent population.

Because of the limited available literature available on our focus subject, our review identified multiple publications by the same group of authors. With the exception of the two research articles that surveyed prison employees across the United States, the majority of our information is geographically restricted our results to California, New York, Rhode Island, Chicago, and a few smaller local jails in the southeast U.S.

## Conclusions

Incarcerated women desire access to standard and emergency contraception from carceral health care systems. Knowledgeable family planning practitioners providing patient-centered and trauma-informed care and public health interventions linking newly released inmates to community clinics can help alleviate inmates’ concerns regarding initiating desired contraception while incarcerated. Access to abortion should be viewed as medically necessary and care coordinated with an outside provider as soon as possible.

## Data Availability

Search terms are provided in the methods and references in each section to allow investigators to gather the same set of articles used in this systematic review.

## References

[1] Glaze LE, Kaeble D. Correctional Populations in the United States, 2015: Bureau of Justice Statistics; 2016. Contract No.: NCJ 250374.

[2] Kajstura A (2018). Women’s mass incarceration: the whole pie 2018.

[3] Sufrin C, Beal L, Clarke J, Jones R, Mosher WD (2019). Pregnancy outcomes in US prisons, 2016–2017. Am J Public Health.

[4] ACOG Committee on Health Care for Underserved Women (2012). Committee opinion 535: reproductive health care for incarcerated women and adolescent females. Obstet Gynecol.

[5] APHA (2003). Task force on correctional health care standards. Standards for health Services in Correctional Institutions. Third ed.

[6] NCCHC (2014). Position Statement: Women’s Health Care in Correctional Settings.

[7] Clarke JG, Hebert MR, Rosengard C, Rose J, DaSilva K, Stein M (2006). Reproductive health care and family planning needs among incarcerated women. Am J Public Health.

[8] Clarke JG, Rosengard C, Rose J, Hebert MR, Phipps MG, Stein MD (2006). Pregnancy attitudes and contraceptive plans among women entering jail. Womens Health.

[9] Clarke JG, Rosengard C, Rose JS, Hebert MR, Peipert J, Stein MD (2006). Improving birth control service utilization by offering services prerelease vs postincarceration. Am J Public Health.

[10] Sufrin CB, Creinin MD, Chang JC (2009). Contraception services for incarcerated women: a national survey of correctional health providers. Contraception.

[11] Sufrin CB, Creinin MD, Chang JC (2009). Incarcerated women and abortion provision: a survey of correctional health providers. Perspect Sex Reprod Health.

[12] Finer LB, Zolna MR (2016). Declines in unintended pregnancy in the United States, 2008–2011. N Engl J Med.

[13] Jones RK, Jerman J (2017). Population group abortion rates and lifetime incidence of abortion: United States, 2008–2014. Am J Public Health.

[14] Hale GJ, Oswalt KL, Cropsey KL, Villalobos GC, Ivey SE, Matthews CA (2009). The contraceptive needs of incarcerated women. J Women's Health.

[15] LaRochelle F, Castro C, Goldenson J, Tulsky JP, Cohan DL, Blumenthal PD (2012). Contraceptive use and barriers to access among newly arrested women. J Correct Health Care.

[16] Cannon R, Madrigal JM, Feldman E, Stempinski-Metoyer K, Holloway L, Patel A (2018). Contraceptive needs among newly incarcerated women in a county jail in the United States. Int J Prison Health.

[17] Fiedler D, Tyler J (1975). Reaching the forgotten: contraception for the institutionalized woman. Adv Plan Parent.

[18] Kasdan D (2009). Abortion access for incarcerated women: are correctional health practices in conflict with constitutional standards?. Perspect Sex Reprod Health.

[19] Guttmacher Institute Public Policy Office. An Overview of Abortion Laws. State Laws and Policies. 2019. Retrieved December 15, 2019, from https://www.guttmacher.org/state-policy/explore/overview-abortion-laws#.

[20] Roth R (2011). Abortion access for imprisoned women: marginalized medical care for a marginalized group. Womens Health Issues.

[21] Sufrin Carolyn, Kolbi-Molinas Alexa, Roth Rachel (2015). Reproductive Justice, Health Disparities And Incarcerated Women in the United States. Perspectives on Sexual and Reproductive Health.

[22] Kouros N (2013). Women inmates in California sterilised without state approval. Monash Bioeth Rev.

[23] ACOG Committee on Ethics (2017). Committee opinion 695: sterilization of women: ethical issues and considerations. Obstet Gynecol.

[24] Roth R, Ainsworth SL (2015). If they hand you a paper, you sign it: a call to end the sterilization of women in prison. Hastings Womens LJ.

[25] Kraft-Stolar T (2015). Reproductive injustice: the state of reproductive health Care for Women in New York state prisons: a report of the women in prison project of the correctional Association of New York: correctional Association of New York.

[26] Sufrin C, Baird S, Clarke J, Feldman E (2017). Family planning services for incarcerated women: models for filling an unmet need. Int J Prison Health.

[27] New York votes to close notorious Rikers Island jail complex. The Guardian. 2019 October 17.

[28] Sufrin C, Oxnard T, Goldenson J, Simonson K, Jackson A (2015). Long-acting reversible contraceptives for incarcerated women: feasibility and safety of on-site provision. Perspect Sex Reprod Health.

[29] Sufrin CB, Tulsky JP, Goldenson J, Winter KS, Cohan DL (2010). Emergency contraception for newly arrested women: evidence for an unrecognized public health opportunity. J Urban Health.

[30] Schonberg D, Bennett AH, Sufrin C, Karasz A, Gold M (2015). What women want: a qualitative study of contraception in jail. Am J Public Health.

[31] Oswalt K, Hale GJ, Cropsey KL, Villalobos GC, Ivey SE, Matthews CA (2010). The contraceptive needs for STD protection among women in jail. Health Educ Behav.

[32] Knittel AK (2019). Resolving health disparities for women involved in the criminal justice system. N C Med J.

[33] Zheng Z (2018). Jail inmates in 2016. US Department of Justice: Office of Justice Programs, Bureau of Justice Statistics.

[34] Carson EA (2018). Prisoners in 2016. Bureau of Justice Statistics Bulletin.

